# Prevalence of dyslipidemia and its association with opium consumption in the Rafsanjan cohort study

**DOI:** 10.1038/s41598-022-13926-3

**Published:** 2022-07-07

**Authors:** Zahra Jamali, Mojgan Noroozi Karimabad, Parvin Khalili, Tabandeh Sadeghi, Ahmadreza Sayadi, Faegheh Mohammadakbari Rostamabadi, Carlo La Vecchia, Ali Esmaeili-Nadimi

**Affiliations:** 1 Non-Communicable Diseases Research Center, Rafsanjan University of Medical Sciences, Rafsanjan, Iran; 2 Molecular Medicine Research Center, Research Institute of Basic Medical Sciences, Rafsanjan University of Medical Sciences, Rafsanjan, Iran; 3 Department of Epidemiology, School of Public Health, Social Determinants of Health Research Centre, Rafsanjan University of Medical Sciences, Rafsanjan, Iran; 4 Department of Pediatric Nursing, School of Nursing and Midwifery, Non‑Communicable Diseases Research Center, Rafsanjan University of Medical Sciences, Rafsanjan, Iran; 5 Department of Psychiatric Nursing, School of Nursing and Midwifery, Social Determinants of Health Research Center, Rafsanjan University of Medical Sciences, Rafsanjan, Iran; 6 Department of Clinical Sciences and Community Health, Università Degli Studi Di Milano, 20133 Milan, Italy

**Keywords:** Diseases, Medical research

## Abstract

The potential effects of opium consumption on lipid profile remain unquantified. We considered the association between opium use and dyslipidemia. In this cross-sectional study, we used data obtained from the Rafsanjan cohort study, as a part of the prospective epidemiological research studies in IrAN (PERSIAN) with detailed and validated data on opium consumption and selected other exposures. A total of 9932 adults were included in the study. Logistic regression models were used to assess the relationships of opium consumption with the prevalence of dyslipidemia and lipid disorders. In this population, 73.33% had dyslipidemia and the prevalence rates of high TC, high TG, high LDL and low HDL were 54.24%, 47.45%, 34.43% and 11.91% respectively. After adjustment for all confounders, opium users compared with non-users had lower odds ratios (OR) of high TC and high LDL [0.81 (95% confidence interval, CI 0.71–0.92) and 0.80 (95% CI 0.69–0.93) respectively] and greater OR of low HDL [1.30 (95% CI 1.04–1.62)]. Longer duration of opium consumption resulted in lower ORs of high TC, 0.68 (95% CI 0.55–0.84) and high LDL, 0.82 (95% CI 0.67–0.99), and shorter duration of opium consumption resulted in increased odds of low HDL, 1.30 (95% CI 1.02–1.66). High dose of opium consumption was associated with an OR of dyslipidemia of 0.80 (95% CI 0.65–0.97), high TC of 0.80 (95% CI 0.67–0.95), and high LDL of 0.78 (95% CI 0.64–0.96) and low dose of opium consumption, with an OR of low HDL of 1.30 (95% CI 1.02–1.65). In relation to route of consumption, opium smoking was a risk factor for low HDL with an adjusted odds ratio of 1.31 (1.04–1.63). Opium use was associated with selected changes on serum lipid levels, but opium users had higher frequency of cardiovascular disease history.

## Introduction

Cardiovascular disease (CVD) is one of the major causes of death worldwide, and its prevalence is increasing because the major risk factors of CVD such as hypertension, diabetes, obesity, and dyslipidemia are rising^1,2^. A total of 40–45% of deaths is attributed to CVD^3^. Atherosclerosis is the main cause of CVD and dyslipidemia is one of the important modifiable risk factors^4^. Proper controlling of lipid levels can reduce the risk of CVD^5^. Successfully management of dyslipidemia risk factors are important in attaining this goal^6^.

Opium is traditionally used in many South and central Asian countries, including Iran. Iran is one of the most important markets for opium due to the easy access to this substance^7^ and disbelief about its role on diabetes mellitus, insulin resistance, and lipid profile disturbances^8^. A previous study reported that Kerman an eastern province of Iran, had the highest opium usage in this country^9^. Opium is likely to alter the blood lipid levels^10–12^. There are many controversies about opium effects on lipid profile and risk of CVD. Previous reports show that adults with opium usage have higher risks of acute myocardial infarction, atherosclerosis, and cardiovascular mortality^13–15^. In contrast, Marmor et al. showed that the usage of opium or morphine could have a favorable effect against cardiovascular diseases^16^.

Some surveys have revealed that opium using elevates total cholesterol (TC), LDL cholesterol (LDL), triglyceride (TG), but HDL cholesterol (HDL) levels remained unchanged^10^. Other studies have reported that opium decreases TC, LDL and TG^11^. On the contrary, in some clinical studies, no significant relationship was seen between opium with TG, HDL and LDL levels^12^.

Prevalence of dyslipidemia is rising in many countries including Iran^17^. Recent research showed that the prevalence of dyslipidemia was 83.4% among Iranian adults^1^. Serum lipid levels differ according to ethnic, social, economic and cultural characteristics in different communities^3,6,18,19^. Awareness of the dyslipidemia prevalence is essential for proper planning of prevention programs. Considering the high rate of dyslipidemia in Iran and given that opium is traditionally used in our country^7^, the present study aimed to assess the prevalence of dyslipidemia and its association with opium consumption in Rafsanjan adult population.

## Methods

### The study population

This study was derived from the recruitment phase of Rafsanjan cohort study (RCS); as part of the prospective epidemiological research studies in IrAN (PERSIAN)^20^. The recruitment phase began in August 2015 in Rafsanjan, a region in the southeast of Iran.

Age range was 35–70 years. The target population (n = 10,000) was reached in December 2017 after 14,827 individuals were recruited to participate. A total number of 9991 subjects participated and signed the written informed consent letter. There were less than 2% of missing values for all variables and because missing values were random (MCAR), we use complete case analysis to handle missing data. Finally 9932 subjects (46.57% male and 53.43% female) were eligible to enter this study. For further details refer to the protocol and research guide^21^. Study protocol was designed according to the Persian cohort study and was approved by the Ethics Committee of Rafsanjan University of Medical Sciences (Ethical codes: ID: IR.RUMS.REC.1399.196).

### Definition and measurements

Participants were interviewed using a standardized questionnaire containing questions on socio-demographic status, smoking, physical activity and other variables related to this study. Questionnaires were validated in the PERSIAN cohort study^20^. Fasting serum TC, TG, HDL, and LDL were measured using a biotecnica analyzer (BT 1500, Italy) at the Central Laboratory in Cohort center. Accuracy and precision of all methods were performed in accordance with the relevant guidelines and regulations.

According to the Third Report of the National Cholesterol Education Program (NCEP-Adult Treatment Panel III), dyslipidemia was defined as LDL ≥ 130 mg/dL, or TC ≥ 200 mg/dL, or HDL ≤ 40 mg/dL in men, and 50 mg/dl in women or TG ≥ 150 mg/dL and or use of lipid-lowering medications in the past two weeks^22^. Furthermore, high LDL, high TC, high TG and low HDL disorders were defined as LDL ≥ 130 mg/dL or use of lipid-lowering medications for high LDL, TC ≥ 200 mg/dL or use of lipid-lowering medications for high TC, TG ≥ 150 mg/dL or use of lipid-lowering medications for high TG, HDL ≤ 40 mg/dL in men, and 50 mg/dl in women respectively.

Smoking and opium usage were self-reported. A participant is defined as an opium user if he/she reported consumption of opium at least once per week for 6 months, prior to admission^15^. To assess opium use, we used a structured questionnaire in which detailed questions about age at the time of starting opium use, opium dose, duration and frequency of use, administration routes (smoking and oral consumption), opium types (teriak, Sukhteh, Shireh, heroin,.) and age at the time of quitting for those who had quitted opium use. Duration of opium use included the number of years the participant used opium throughout the participant’s life. Opium dose was defined as the dose of opium use throughout participant’s life (dose-year: the number of years the participant used opium once per day). Smoking was classified into nonsmoker, current smoker and former smoker. The Socio-Economic Status (SES) of individuals was also determined using the Wealth score index (WSI). WSI was calculated by multiple correspondence analysis (MCA) of the subjects’ economic and social variables^23^. Then the scores were collected for each individual. After this step, the subjects were categorized into quartiles.

Body Mass Index (BMI) was calculated by dividing weight (kg) by squared height (m2) and was classified into BMI < 25, 25.0 ≤ BMI < 30, and BMI ≥ 30^15^. A validated Food Frequency Questionnaire (FFQ) was filled out to evaluate fat intake (grams per day) in the participants of this study. To evaluate the level of physical activity, metabolic equivalent of task (MET) was used. Physical activity was assessed based on a 22-item questionnaire and the daily physical activity. Individuals categorized as low (≤ 35.29 MET-hours per week), moderate (35.30–40.32 MET-hours per week) and heavy (≥ 40.32 MET-hours per week) groups based on the 25th and 75th percentile. The variables such as age, gender, BMI, physical activity, education, wealth status index, cigarette smoking, alcohol drinking, diabetes (yes/no), hypertension (yes/no), hepatitis (yes/no), renal failure (yes/no), total fat intake (continuous variable), use of hepatotoxic drugs (yes/no) and fatty liver (yes/no) were considered as covariates. Opium consumption (yes, no), duration and dose of use and administration routes (smoking and oral consumption) were the independent variables. Dyslipidemia, high LDL, high TC, high TG and low HDL disorders were considered as the dependent variables.

### Statistical analyses

Comparisons were made between participants with dyslipidemia and non-dyslipidemia using the chi square test for categorical variables, t test for normally distributed quantitative variables and the Mann Whitney U Test for non-normally distributed quantitative variables. Univariate logistic regression was performed to identify the factors associated with lipid disorders in the study participants. Logistic regression models were used to investigate the association between opium use and the prevalence of dyslipidemia and other lipid disorders. Potential confounders were sequentially entered into models according to their hypothesized strengths of association with opium use and lipid disorders. To reach this goal, separate models at bivariate level were run to obtain variables associated with lipid disorders. Variables with a p-value < 0.25 were considered for multivariate analysis. Adjusted model 1 included basic socio-demographic characteristics (age, gender, education years and WSI) considered to be the most strongly related to both opium use and lipid disorders. Adjusted model 2 adjusted for lifestyle confounding variables (cigarette smoking, alcohol drinking), BMI and physical activity level in addition to the socio-demographic characteristics. Adjusted model 3 included all variables in adjusted model 2 and additionally included for diabetes, hypertension, hepatitis, renal failure, total fat intake, use of hepatotoxic drugs and fatty liver. In all models, variables of age, education years, BMI, physical activity level and total fat intake were entered continuously. For opium users, duration and dose of opium consumption were categorized into two groups based on mean to test for dose response association. Also, the data were analyzed by route of opium consumption. All analyses were conducted in State V.12. All p-values are two-sided, and p-values < 0.05 and 95% confidence intervals were considered as significant.

### Ethics approval and consent to participate

The ethics committee of Rafsanjan University of Medical Sciences approved this study (Ethical codes: ID: IR.RUMS.REC.1399.196). Written informed consent was obtained from the participants. The data of participants kept confidential and was only accessible to the study investigators.

## Results

### Demographic, selected medical and laboratory characteristics of study participants

**Figure 1:**
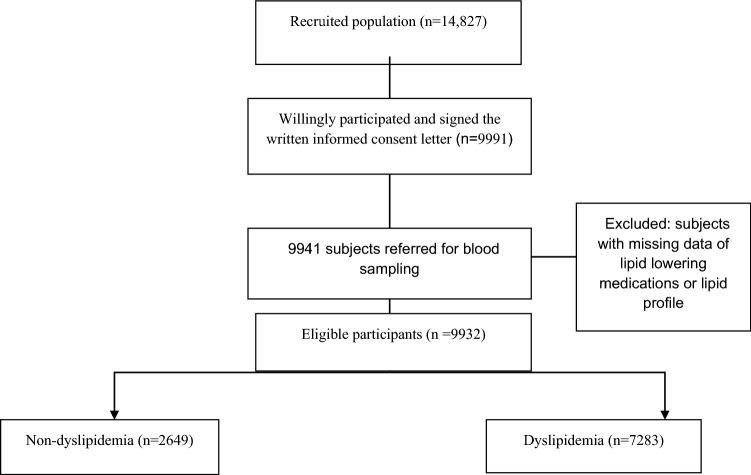
Flowchart of the study design of lipid profile in Rafsanjan cohort study.

Figure 1 shows the flow chart of the study design of lipid profile in Rafsanjan cohort study. A total of 9932 subjects (46.57% male and 53.43% female) were eligible to enter the study in this research, among whom 7283 (73.33%) had dyslipidemia, including (44.90% of the men and 55.10% of the women. As seen in Table 1, 1667 (16.90%) were current smokers and 2324 (23.56%) were opium users. Age, gender, education level, marital status, occupation, BMI, physical activity, cigarette smoking, alcohol and opium consumption, dose, duration and route of opium consumption, use of hepatotoxic drugs, history of hypertension, diabetes, fatty liver, renal failure and CVD history had significant relationships with dyslipidemia. However, WSI was not a significant variable for dyslipidemia. The mean levels of TC, TG, LDL, age and BMI were significantly higher in participants with dyslipidemia than those in the non-dyslipidemia participants, whereas the mean levels of physical activity, education years, and HDL were lower in dyslipidemic subjects.

**Table 1:** Demographic, selected medical and laboratory characteristics of study participants (n = 9932).

Characteristics	All (n = 9932)	Non-dyslipidemia (n = 2649)	Dyslipidemia (n = 7283)	*p* value
**Age—yr—no. (%)**				< 0.001
35–45	3695 (37.20)	1282 (48.40)	2413 (33.13)	
46–55	3055 (30.56)	741 (27.97)	2314 (31.77)	
≥ 56	3182 (32.04)	626 (23.63)	2556 (35.10)	
Mean ± SD	49.94 ± 9.56	47.04 ± 9.68	50.46 ± 9.45	< 0.001
**Gender—no. (%)**				< 0.001
Female	5307 (53.43)	1294 (48.85)	4013 (55.10)	
Male	4625 (46.57)	1355 (51.15)	3270 (44.90)	
**Education—no. (%)**				< 0.001
≤ 5 years	3478 (35.05)	784 (29.62)	2694 (37.02)	
6–12 years	4811 (48.48)	1385 (52.32)	3426 (47.08)	
≥ 13 years	1635 (16.48)	478 (18.06)	1157 (15.90)	
**Physical activity—no (%)**				< 0.001
Low	2540 (25.57)	571 (21.56)	1969 (27.04)	
Moderate	4912 (49.46)	1239 (46.77)	3673 (50.43)	
Heavy	2480 (24.97)	839 (31.67)	1641 (22.53)	
Mean ± SD	38.77 ± 6.35	40.01 ± 7.28	38.55 ± 6.14	< 0.001
**BMI—no. (%)**				
< 25	2862 (28.84)	1199 (45.31)	1663 (22.85)	
25–29.9	4069 (41.00)	925 (34.96)	3144 (43.19)	
≥ 30	2994 (30.17)	522 (19.73)	2472 (33.96)	
Mean ± SD	27.83 ± 4.92	25.64 ± 5.02	28.21 ± 4.81	< 0.001
**Wealth score index—no (%)**			0.072	
Low	2321 (23.39)	646 (24.42)	1675 (23.02)	
Low-middle	2848 (28.71	708 (26.77)	2140 (29.41)	
Middle-high	3980 (40.12)	1082 (40.91)	2898 (39.83)	
High	772 (7.78)	209 (7.90)	563 (7.74)	
**Marital status—no. (%)**				< 0.001
Single	780 (7.85)	160 (6.04)	620 (8.51)	
Married	9152 (92.15)	2489 (93.96)	6663 (91.49)	
**Alcohol consumption—no. (%)**				0.029
Yes	985 (9.99)	291 (11.08)	694 (9.59)	
No	8878 (90.01)	2335 (88.92)	6543 (90.41)	
**Cigarette smoking—no. (%)**				< 0.001
Current	1667 (16.90)	533 (20.30)	1134 (15.67)	
Former	862 (8.74)	220 (8.38)	642 (8.87)	
Never	7334 (74.36)	1873 (71.33)	5461 (75.46)	
**Opium consumption—no. (%)**				< 0.001
Yes	2324 (23.56)	696 (26.50)	1628 (22.50)	
No	7539 (76.44)	1930 (73.50)	5609 (77.50)	
**Route of opium consumption—no. (%)**				< 0.001
Non-user	7596 (76.48)	1947 (73.50)	5649 (77.56)	
Smoking	191 (1.92)	71 (2.68)	120 (1.65)	
Oral	2145 (21.60)	631 (23.82)	1514 (20.79)	
**Duration of opium consumption—no. (%)**				< 0.001
Non-user	7627 (76.79)	1962 (74.07)	5665 (77.78)	
Users				
≤ mean	1255 (12.64)	348 (13.14)	907 (12.45)	
> mean	1050 (10.57)	339 (12.80)	711 (9.76)	
**Dose of opium consumption—no. (%)**				< 0.001
Non-user	7627 (76.79)	1962 (74.07)	5665 (77.78)	
Users				
≤ mean	1392 (14.02)	382 (14.42)	1010 (13.87)	
> mean	913 (9.19)	305 (11.51)	608 (8.35)	
**Hypertension—no. (%)**				< 0.001
Yes	2231 (22.57)	324 (12.30)	1907 (26.31)	
No	7652 (77.43)	2311 (87.70)	5341 (73.69)	
**Diabetes—no. (%)**				< 0.001
Yes	2858 (28.93)	448 (17.01)	2410 (33.26)	
No	7021 (71.07)	2185 (82.99)	4836 (66.74)	
**Renal failure—no. (%)**				0.048
Yes	9829 (99.45)	8 (0.30)	46 (0.63)	
No	54 (0.55)	2627 (99.70)	7202 (99.37)	
**Hepatitis—no. (%)**				0.052
Yes	28 (0.28)	12 (0.46)	16 (0.22)	
No	9855 (99.72)	2623 (99.54)	7232 (99.78)	
**CVD history—no. (%)**				< 0.001
Yes	1025 (10.37)	134 (5.09)	891 (12.29)	
No	8858 (89.63)	2501 (94.91)	6357 (87.71)	
**Fatty liver—no. (%)**				< 0.001
Yes	1008 (10.20	168 (6.38)	840 (11.59)	
No	8875 (89.80)	2467 (93.62)	6408 (88.41)	
**Taking hepatotoxic drugs—no. (%)**				< 0.001
Yes	2378 (24.06)	266 (10.09)	2112 (29.14)	
No	7505 (75.94)	2369 (89.91)	5136 (70.86)	
**Total fat intake—no. (%)**				0.001
≤ mean	5720 (57.88)	1453 (55.04)	4267 (58.91)	
> mean	4163 (42.12)	1187 (44.96)	2976 (41.09)	
Mean ± SD	55.70 ± 23.65	57.36 ± 24.18	55.10 ± 23.43	0.001
**Cholesterol**				< 0.001
Mean ± SD	198.66 ± 38.07	162.84 ± 17.32	205.02 ± 37.22	
**Triglycerides**				< 0.001
Median (interquartile range)	145 (106–199)	100 (80–122)	170 (129–225)	
**HDL cholesterol**				< 0.001
Mean ± SD	57.75 ± 10.88	59.59 ± 9.99	57.42 ± 11.00	
**LDL cholesterol**				< 0.001
Mean ± SD	108.18 ± 30.31	83.68 ± 12.41	112.54 ± 30.48	

### Association between serum high TC, high TG, low HDL and high LDL with selected variables in study participants

Table 2 describes the association between serum high TC, high TG, low HDL and high LDL with selected variables of study participants. The prevalence rates of high TC, high TG, high LDL and low HDL disorders were 54.24%, 47.45%, 34.43% and 11.91% respectively. High TC and high LDL disorders were significantly higher at the age group of ≥ 56 years while low HDL was significantly observed at the age group of 35–45 years. High TC, low HDL and high LDL disorders were higher in women than men. On the other hand, high TG disorder was more common among men. The lowest rates of high TC, low HDL and high LDL disorders were observed at the group with education level more than 12 years. Participants with BMI < 25 and heavy physical activity had the lowest levels of lipid disorders. Cigarette smoking, alcohol and opium consumption, dose, duration and route of opium consumption, use of hepatotoxic drugs, history of hypertension, diabetes and fatty liver were related to lipid measurements.

**Table 2:** Association between serum high TC, high TG, low HDL and high LDL with selected variables in study participants.

Characteristic	High TC (n = 5394)	Unadjusted OR(95% CI)	*p* value	High TG (n = 4717)	Unadjusted OR(95% CI)	*p* value	Low HDL (n = 1184)	Unadjusted OR(95% CI)	*p* value	High LDL (n = 3420)	Unadjusted OR(95% CI)	*p* value
**Age cat—no. (%)**			< 0.001			0.001			< 0.009			< 0.001
35–45	1482 (27.47)	1		1635 (34.66)	1		486 (41.05)	1		735 (21.49)	1	
46–55	1761 (32.65)	2.03 (1.83–2.23)		1543 (32.71)	1.29 (1.17–1.42)		330 (27.87)	0.80 (0.69–0.93)		1074 (31.40	2.18 (1.96–2.44)	
≥ 56	2151 (39.88)	3.11 (2.82 (3.44)		1539 (32.63)	1.18 (1.07–1.30)		368 (31.08)	0.86 (0.75–0.99)		1611 (47.11)	4.12 (3.7–4.59)	
**Gender—no. (%)**			< 0.001			0.001			0.001			< 0.001
Female	3113 (57.71)	1.46 (1.35–1.58)		2356 (49.95)	0.77 (0.71–0.83)		908 (76.69)	3.25 (2.82–3.74)		2056 (60.12)	1.51 (1.39–1.64)	
Male	2281 (42.29)	1		2361 (50.05)	1		276 (23.31)	1		1364 (39.88)	1	
**Education-no. (%)**			< 0.001			0.063			< 0.001			< 0.001
≤ 5 years	2131 (39.54)	1		1707 (36.23)	1		491 (41.54)	1		1473 (43.08)	1	
6–12 years	2413 (44.77)	0.64 (0.58–0.70)		2235 (47.43)	0.90 (0.83–0.98)		550 (46.53)	0.79 (0.69–0.90)		1444 (42.23)	0.58 (0.53–0.64)	
> 12 years	846 (15.70)	0.68 (0.6–0.77)		770 (16.43)	0.93 (0.82–1.04)		141 (11.93)	0.57 (0.47–0.70)		502 (14.68)	0.60 (0.53–0.68)	
**Physical activity—no. (%)**			< 0.001			< 0.001			< 0.001			< 0.001
Low	1480 (27.44)	1		2027 (42.97)	1		290 (24.49)	1		997 (29.15)	1	
Moderate	2733 (50.67)	0.90 (0.82–0.99)		2254 (47.78)	0.77 (0.70–0.65)		650 (54.90)	1.18 (1.02–1.34)		1701 (49.74)	0.82 (0.74–0.91)	
Heavy	1181 (21.89)	0.65 (0.58–0.73)		436 (9.24)	0.64 (0.57–0.72)		244 (20.61)	0.85 (0.71–1.02)		722 (21.11)	0.64 (0.57–0.72)	
**BMI—no. (%)**			< 0.001			< 0.001			< 0.001			< 0.001
< 25	12.14 (22.52)	1		947 (20.09)	1		205 (17.31)	1		749 (21.91)	1	
25–29.9	2313 (42.90)	1.79 (1.62–1.97)		2101 (44.58)	2.16 (1.95–2.38)		514 (43.41)	1.87 (1.58–2.22)		1454 (42.54)	1.57 (1.41–1.74)	
≥ 30	1864 (34.58)	2.23 (2.01–2.47)		1665 (35.33)	2.52 (2.27–2.81)		465 (39.27)	2.38 (2.00–2.83)		1215 (35.55)	1.93 (1.72–2.15)	
**Wealth score index—no (%)**			0.340			0.349			< 0.001			< 0.001
Low	1280 (23.75)	1		1066 (22.63)	1		318 (26.90)	1		767 (25.36)	1	
Low-middle	1561 (28.96)	0.99 (0.88–1.10)		1377 (29.23)	1.10 (0.99–1.23)		403 (34.09)	1.04 (0.86–1.22)		1033 (30.21)	0.95 (0.85–1.07)	
Middle-high	2119 (39.31)	0.93 (0.84–1.03)		1896 (40.25)	1.07 (0.97–1.19)		415 (35.11)	0.73 (0.63–0.86)		1270 (37.15)	0.79 (0.71–0.87)	
High	430 (7.98)	1.02 (O.87–1.20)		372 (7.90)	1.10 (0.93–1.29)		46 (3.89)	0.40 (0.29–0.55)		249 (7.28)	0.80 (0.67–0.94)	
**Marital status—no. (%)**			< 0.001			0.906			< 0.001			< 0.001
single	500 (9.27)	1		369 (7.82)	1		126 (10.64)	1		358 (10.47)	1	
Married	4894 (90.73)	O.64 (0.55–0.75)		4348 (92.18)	1.01 (0.87–1.16)		1058 (89.36)	0.68 (0.56–0.83)		3062 (89.53)	0.59 (0.51–0.69)	
**Alcohol consumption—no. (%)**			< 0.001			< 0.001			< 0.001			< 0.001
Yes	448 (8.35)	0.67 (0.59–0.77)		520 (11.09)	1.26 (1.11–1.44)		70 (5.97)	0.54 (0.42–0.69)		263 (7.73)	0.67 (0.57–0.77)	
No	4917 (91.65)	1		4167 (88.91)	1		1103 (94.03)	1		3140 (92.27)	1	
**Cigarette smoking—no. (%)**			< 0.001			0.024			< 0.001			< 0.001
Current	763 (14.22)	0.66 (0.59–0.73)		802 (17.11)	1.05 (0.94–1.17)		152 (12.96)	0.67 (0.56–0.80)		463 (13.61)	0.69 (0.61–0.77)	
Former	480 (8.95)	0.98 (0.85–1.13)		446 (9.52)	1.21 (1.05–1.40)		62 (5.29)	0.52 (0.39–0.67)		311 (9.14)	0.01 (0.87–1.17)	
Never	4129 (76.83)	1		3439 (73.57)	1		959 (81.76)	1		2629 (77.26)	1	
**Opium consumption—no. (%)**			0.001			0.007			< 0.001			< 0.001
Yes	1134 (21.14)	0.74 (0.68–0.81)		1162 (24.79)	1.14 (1.04–1.25)		206 (17.56)	0.66 (0.56–0.77)		710 (20.86)	0.79 (0.72–0.88)	
No	4231 (78.86)	1		3525 (75.21)	1		967 (82.44)	1		2693 (76.14)	1	
**Route of opium consumption—no. (%)**			0.002			< 0.001			< 0.001			< 0.001
Non-user	4256 (78.90)	1		3551 (75.28)	1		975 (82.35)	1		2708 (79.18)	1	
smoking	1054 (19.54)	0.76 (0.69–0.83)		1086 (23.02)	1.17 (1.06–1.28)		192 (16.22)	0.67 (0.57–0.79)		649 (18.98)	0.78 (0.71–1.221)	
oral	84 (1.56)	0.60 (0.45–0.8)		80 (1.70)	0.81 (0.61–1.09)		17 (1.44)	0.66 (0.40–1.09)		63 (1.84)	0.89 (0.65–1.21)	
**Duration of opium consumption—no. (%)**			< 0.001			< 0.001			< 0.001			< 0.001
Non-user	4269 (79.14)	1		3560 (75.47)	1		978 (82.60)	1		2715 (79.39)	1	
Users												
≤ mean	624 (11.57)	0.77 (0.69–0.87)		667 (14.14)	1.29 (1.14–1.45)		119 (10.05)	0.71 (0.58–0.87)		384 (11.23)	0.80 (0.70–0.91)	
> mean	501 (9.29)	0.72 (0.63–0.82)		490 (10.39)	1.00 (0.88–1.14)		87 (7.35)	0.61 (0.49–0.77)		321 (9.39)	0.80 (0.69–0.91)	
**Dose of opium consumption—no. (%)**			< 0.001			< 0.001			< 0.001			< 0.001
Non-user	4269 (79.14)	1		3560 (75.47)	1		978 (82.60)	1		2715 (79.39)	1	
Users												
≤ mean	700 (12.98)	0.79 (0.71–0.89)		733 (15.54)	1.26 (1.12–1.42)		119 (10.05)	0.70 (0.58–0.85)		428 (12.51)	0.80 (0.71–0.91)	
> mean	425 (7.88)	0.72 (0.63–0.82)		424 (8.99)	0.99 (0.86–1.14)		87 (7.35)	0.61 (0.48–0.78)		277 (8.10)	0.79 (0.68–0.91)	
**Hypertension—no. (%)**			< 0.001			< 0.001			< 0.001			< 0.001
Yes	1549 (28.83)	2.27 (2.05–2.51)		1236 (26.34)	1.50 (1.37–1.65)		352 (29.91)	1.55 (1.35–1.77)		1162 (34.11)	2.62 (2.38–2.88)	
No	3824 (71.17)	1		3457 (73.66)	1		825 (70.09)	1		2245 (65.89)	1	
**Diabetes—no. (%)**		< 0.001			< 0.001			< 0.001			< 0.001	
Yes	1370 (25.50)	2.42 (2.17–2.69)		1155 (24.61)	1.86 (1.68–2.06)		299 (25.40)	1.42 (1.18–1.70)		1021 (29.97)	2.633 (2.38–2.91)	
No	4003 (74.50)	1		3538 (75.39)	1		878 (74.60)	1		2386 (70.03)	1	
**Fatty liver—no. (%)**			< 0.001			< 0.001			< 0.001			< 0.001
Yes	629 (11.71)	1.44 (1.26–1.65)		585 (12.47)	1.60 (1.40–1.82)		157 (13.34)	1.42 (1.18–1.70)		427 (12.53)	1.45 (1.27–1.66)	
No	4744 (88.29)	1		4108 (87.53)	1		1020 (86.66)	1		2980 (87.47)	1	
**Taking hepatotoxic drugs—no. (%)**			< 0.001			< 0.001			< 0.001			< 0.001
Yes	1860 (34.62)	4.04 (3.63–4.50)		1244 (26.51)	1.29 (1.17–1.41)		348 (29.57)	1.38 (1.21–1.59)		1585 (46.52)	6.24 (5.64–6.89)	
No	3513 (65.38)	1		3449 (73.49)	1		829 (70.43)	1		1822 (53.48)	1	
**Renal failure—no. (%)**			0.2			0.022			0.507			0.067
Yes	34 (0.63)	1.43 (0.82–2.49)		34 (0.72)	1.89 (1.09–3.29)		8 (0.68)	1.29 (0.61–2.74)		25 (0.37)	1.64 (0.96–2.81)	
No	5339 (99.37)			4659 (99.28)	1		1169 (99.32)	1		3382 (99.27)	1	
**Hepatitis—no. (%)**			0.11			0.045			0.329			0.291
Yes	11 (0.2)	0.54 (0.25–1.16)		8 (0.17)	0.44 (0.19–1.00		5 (0.42)	1.61 (0.61–4.23)		7 (0.21)	0.63 (0.27–1.49)	
No	5362 (99.80)	1		4685 (99.83)	1		1172 (99.58)	1		3400 (0.21)	1	
**CVD history—no. (%)**			< 0.001			0.608			< 0.001			< 0.001
Yes	777 (14.46)	2.87 (2.47–3.32)		479 (10.21	0.97 (0.85–1.10)		184 (15.63)	1.73 (1.46–2.06)		674 (19.78)	4.30 (3.75–4.93)	
No	4596 (85.54)	1		4214 (89.79)	1		993 (84.37)	1		2733 (80.22)	1	
**Total fat intake—no. (%)**			< 0.001			0.673			0.001			< 0.001
≤ mean	3521 (60.53)	1		2724 (58.11)	1		735 (62.29)	1		2137 (62.72)	1	
> mean	2120 (39.47)	078 (0.73–0.86)		1964 (41.89)	0.98 (0.91–1.06)		445 (37.71)	0.81 (0.72–0.92)		1270 (37.28)	0.74 (0.68–0.80)	

### Association of opium consumption with dyslipidemia, high TC, high TG, low HDL and high LDL

Table 3 presents the association of opium use with dyslipidemia, high TC, high TG, low HDL and high LDL disorders. The odds of dyslipidemia in the crude (OR 0.80, 95% CI 0.73–0.89) and adjusted model 1 (OR 0.82, 95% CI 0.72–0.92) were lower among opium users compared with non-users. However, after adjustment for the variables in adjusted models 2 and 3, there was no significant association of dyslipidemia with opium. In the unadjusted model, the odds of high TG (OR 1.14, 95% CI 1.04–1.25) was greater in opium users compared with non-users. while, after adjustment for all mentioned variables (adjusted models 1, 2 and 3), there was no significant association of high TG with opium use. The odds of high TC and high LDL in the crude model and adjusted model 1 were lower among opium users compared with non-users. This association persisted after adjustment for all confounders (adjusted model 3). The corresponding adjusted ORs calculated for opium users in comparison to non-users were 0.81 (95% CI 0.71–0.92) and 0.80 (95% CI 0.69–0.93) respectively, for high TC and high LDL. The odds of low HDL in all adjusted models was significantly higher among opium users compared with non-users.

**Table 3:** Association of opium consumption with Dyslipidemia, High TC, High TG, Low HDL and High LDL in study participants (n = 9932).

	Crude model	Adjusted model 1	Adjusted model 2	Adjusted model 3
	OR (95% CI)^a^	OR (95% CI)^b^	OR (95% CI)^c^	OR (95% CI)^d^
**Dyslipidemia**				
**Opium consumption**				
No	1	1	1	1
Yes	0.80 (0.73–0.89)	0.82 (0.72–0.92)	0.91 (0.78–1.05)	0.89 (0.77–1.03)
**Route of opium consumption**				
Non-user	1	1	1	1
Smoking	0.83 (0.74–0.92)	0.84 (0.74–0.96)	0.85 (0.72–1.01)	0.92 (0.79–1.07)
Oral	0.58 (0.43–0.78)	0.54 (0.39–0.73)	0.57 (0.39–0.82)	0.60 (0.43–0.85)
**Duration of opium consumption**				
Non-user	1	1	1	1
≤ mean	0.90 (0.79–1.03)	0.92 (0.80–1.07)	0.96 (0.81–1.13)	0.94 (0.80–1.12)
> mean	0.73 (0.63–0.83)	0.73 (0.62–0.85)	0.86 (0.71–1.03)	0.85 (0.70–1.03)
**Dose of opium consumption**				
Non-user	1	1	1	1
≤ mean	0.92 (0.81–1.04)	0.94 (0.82–1.09)	0.98 (0.84–1.16)	0.97 (0.82–1.14)
> mean	0.69 (0.60–0.80)	0.68 (0.57–0.80)	0.80 (0.66–0.97)	0.80 (0.65–0.97)
**High TC**				
**Opium consumption**				
No	1	1	1	1
Yes	0.74 (0.68–0.81)	0.78 (0.70–0.87)	0.84 (0.73–0.95)	0.81 (0.71–0.92)
**Route of opium consumption**				
Non-user	1	1	1	1
Smoking	0.76 (0.69–0.83)	0.80 (0.72–0.90)	0.84 (0.74–0.96)	0.83 (0.72–0.95)
Oral	0.60 (0.45–0.80)	0.54 (0.40–0.74)	0.61 (0.44–0.84)	0.55 (0.40–0.77)
**Duration of opium consumption**				
Non-user	1	1	1	1
≤ mean	0.77 (0.69–0.87)	0.81 (0.71–0.93)	0.84 (0.73–0.97)	0.76 (0.62–0.92)
> mean	0.72 (0.63–082)	0.75 (0.64–0.87)	0.84 (0.71–0.99)	0.68 (0.55–0.84)
**Dose of opium consumption**				
Non-user	1	1	1	1
≤ mean	0.79 (0.71–0.89)	0.84 (0.74–0.96)	0.87 (0.76–1.00)	0.81 (0.70–0.94)
> mean	0.68 (0.60–0.79)	0.69 (0.59–0.81)	0.77 (0.65–0.92)	0.80 (0.67–0.95)
**High TG**				
**Opium consumption**				
No	1	1	1	1
Yes	1.14 (1.04–1.25)	0.93 (0.84–1.04)	1.02 (0.87–1.21)	1.06 (0.93–1.21)
**Route of opium consumption**				
Non-user	1	1	1	1
Smoking	1.17 (1.06–1.28)	0.96 (0.86–1.07)	1.06 (0.90–1.25)	1.08 (0.95–1.24)
Oral	0.81 (0.61–1.09)	0.66 (0.49.89)	0.72 (0.48–1.08)	0.81 (0.59–1.12)
**Duration of opium consumption**				
Non-user	1	1	1	1
≤ mean	1.29 (1.15–1.46)	1.08 (0.94–1.23)	1.12 (0.97–1.29)	1.14 (0.98–1.31)
> mean	1.00 (0.88–1.14)	0.81 (0.70–0.93)	0.94 (0.80–1.11)	0.97 (0.82–1.15)
**Dose of opium consumption**				
Non-user	1	1	1	1
≤ mean	1.27 (1.13–1.42)	1.05 (0.93–1.20)	1.10 (0.96–1.27)	1.12 (0.97–1.29)
> mean	1.00 (0.87–1.14)	0.80 (0.69–0.93)	0.95 (0.80–1.13)	0.98 (082–1.17)
**Low HDL**				
**Opium consumption**				
No	1	1	1	1
Yes	0.66 (0.56–0.77)	1.53 (1.25–1.87)	1.29 (1.04–1.62)	1.30 (1.04–1.62)
**Route of opium consumption**				
Non-user	1	1	1	1
Smoking	0.67 (0.57–0.79)	1.55 (1.26–1.90)	1.37 (1.08–1.74)	1.31 (1.04–1.63)
Oral	0.66 (0.40–1.09)	1.48 (0.88–2.51)	1.29 (0.71–2.37)	1.23 (0.71–2.12)
**Duration of opium consumption**				
Non-user	1	1	1	1
≤ mean	0.71 (0.58–0.87)	1.53 (1.22–1.92)	1.31 (1.03–1.67)	1.30 (1.02–1.66)
> mean	0.61 (0.49–0.77)	1.55 (1.18–2.03)	1.29 (0.95–1.73)	1.30 (0.98–1.76)
**Dose of opium consumption**				
Non-user	1	1	1	1
≤ mean	0.70 (0.58–0.85)	1.53 (1.22–1.91)	1.30 (1.03–1.66)	1.30 (1.02–1.65)
> mean	0.61 (0.48–0.78)	1.55 (1.17–2.07)	1.30 (0.95–1.78)	1.32 (0.97–1.81)
**High LDL**				
**Opium consumption**				
No	1	1	1	1
Yes	0.79 (0.72–0.78)	0.86 (0.76–0.97)	0.88 (0.77–1.00)	0.80 (0.69–0.93)
**Route of opium consumption**				
Non-user	1	1	1	1
Smoking	0.78 (0.71–0.87)	0.85 (0.75–0.97)	0.88 (0.76–1.01)	0.81 (0.70–0.94)
Oral	0.89 (0.65–1.21)	0.81 (0.59–1.12)	0.89 (0.89–1.24)	0.75 (0.52–1.07)
**Duration of opium consumption**				
No user	1	1	1	1
≤ mean	0.80 (0.70–0.91)	0.86 (0.74–1.01)	0.87 (0.74–1.02)	0.79 (0.67–0.93)
> mean	0.80 (0.69–0.92)	0.85 (0.72–1.01)	0.91 (0.76–1.09)	0.82 (0.67–0.99)
**Dose of opium consumption**				
No user	1	1	1	1
≤ mean	0.80 (0.71–0.91)	0.88 (0.76–1.01)	0.89 (0.76–1.04)	0.81 (0.69–0.95)
> mean	0.79 (0.68–0.91)	0.82 (0.69–0.97)	0.87 (0.72–1.05)	0.78 (0.64–0.96)

^a^The baseline model is stratified on the status of opium consumption.

^b^The adjusted model 1 is adjusted for confounding variables age (continuous variable), gender (male/ female), education years (continuous variable) and wealth status index.

^C^The adjusted model 2 has additional adjustment for confounding variables related to lifestyle (cigarette smoking and alcohol drinking), body mass index (continuous variable) and physical activity level (continuous variable).

^d^The adjusted model 3 has additional adjustment for diabetes (yes/no), hypertension (yes/no), hepatitis (yes/no), renal failure (yes/no), total fat intake (continuous variable), taking hepatotoxic drugs (yes/no) and fatty liver (yes/no).

The results were divided into two groups by the mean consumption duration in the opium users. The mean consumption duration was 14.98 ± 10.05 years. After adjustment for all confounders, consumption duration of opium above the mean resulted in lower odds of high TC 0.68 (95% CI 0.55–0.84), and high LDL 0.82 (95% CI 0.67–0.99). Also, consumption duration of opium below the mean resulted in increased odds of low HDL 1.30 (95% CI 1.02–1.66).

The mean opium dosage in the current users was 19.48 ± 23.24. Dose of opium consumption over the mean resulted in an OR of dyslipidemia of 0.80 (95% CI 0.65–0.97), high TC 0.80 (95% CI 0.67–0.95), and high LDL 0.78 (95% CI 0.64–0.96) in the full adjusted model. Furthermore, dose of opium consumption below the mean resulted in increased odds of low HDL 1.30 (95% CI 1.02–1.65).

With reference to consumption route, opium smoking was unrelated to high TC and high LDL disorders with an full adjusted OR of 0.83 (95% CI 0.72–0.95) and 0.81 (95% CI 0.70–0.94) respectively, but this route was associated to low HDL with an full adjusted OR of 1.31 (95% CI 1.04–1.63). Oral administration was inversely related to dyslipidemia and high TC with an adjusted ORs of 0.60 (95% CI 0.43–0.85) and 0.55 (95% CI 0.40–0.77) respectively.

We compared the frequency of subjects with CVD history between opium users and non-users. There was a significant difference regarding CVD history between opium users (15.27%) and non-users (8.87%). Furthermore, to assess the association of opium consumption with dyslipidemia, high TC, high TG, low HDL and high LDL in participants with CVD history, a subgroup analysis of the study population according to the presence or absence of cardiovascular disease (CVD) was done. In the fully adjusted model in participants without CVD history, opium addicts compared to non-addicts significantly had lower odds of high TC (OR 0.83, 95% CI 0.71–0.95) and high LDL (OR 0.76, 95% CI 0.65–0.89). Furthermore, in participants with CVD history, the odds of dyslipidemia (OR 0.43, 95% CI 0.24–0.76) and high TC (OR 0.54, 95% CI 0.35–0.84) was lower and the odds of high TG (OR 1.45, 95% CI 1.01–2.10) was greater in opium users compared with non-users (eTable 1).

## Discussion

This is a cross-sectional study aimed at assessment of the association between opium use and dyslipidemia in the participants of the Rafsanjan Cohort Study, an area in southeast of Iran with a relatively high prevalence of opium use. Age, gender, education level, marital status, occupation, BMI, physical activity, cigarette smoking, alcohol and opium consumption, use of hepatotoxic drugs, history of hypertension, diabetes, fatty liver, renal failure and CVD history showed significant relationships with dyslipidemia.

The prevalence of dyslipidemia was close to the results of other studies reported among Iranian adults^1,24^. The prevalence of dyslipidemia in the other studies reported in the literature varied between 14 and 83%^1,25^. This variation could be due to differences in their race, cultures, lifestyle habits, socioeconomic status, environmental factors, age range and the different definitions of dyslipidemia based on cut-off values for lipid components.

Overall, 2324 (23.56%) of participants were opium users. This was similar to a cohort study on the population of Fasa in the South of Iran that reported 24.1% of the participants used opium^22^. However, the prevalence of opium use in our society was higher than other studies^26,27^. The considerable causes for the high prevalence of opium abuse in this study may be the location of Rafsanjan city in the route of transportation of opium into Iran^28^. Furthermore, in our society, people believe that consumption of opium is effective in controlling blood pressure, lipid disorders, diabetes and heart diseases^16^.

The main finding of this study was that there was a direct association between opium use and a decreased odds of high TC and high LDL and an increased odds of low HDL even after adjustment for potential confounding variables such as those related to demographic, lifestyle, history of diabetes, hypertension, hepatitis, renal failure, and fatty liver, total fat intake and use of hepatotoxic drugs. This is consistent with previous reports among similar population groups^12,22,29^. A study reported that opium users had lower levels of TC, LDL, and HDL in comparison with non-users by including some confounders such as age, gender, BMI, fat intake, medications, smoking and alcohol consumption in the logistic regression model. But opium usage had no correlation with the TG level^22^. Gozashti et al. showed that TC and HDL significantly decreased in opium addicts, but TG remained unchanged^29^. A study by Fatemi et al. confirmed that TC significantly reduced in people consuming opium^12^.

Several studies on healthy subjects have indicated no significant relations between opium addiction and TG, TC, HDL and LDL levels^8,30–32^. On the other hand, some studies have shown that opium addiction has a detrimental effect on one or more lipid components^33–35^. In a study on male cigarette smokers, HDL was lower in opium users compared with non users^33^. In another study, serum levels of TG, TC, and LDL in opium addicted individuals were significantly higher than non-addicted individuals^35^. Furthermore, in study on patients candidates for coronary artery bypass grafting, opium addicts significantly had higher levels of LDL and TG compared to non-addicts^34^. In agreement with this study in our study among participants with CVD history, in opium users compared with non-users, the odds of high TG was greater, while the odds of dyslipidemia and high TC was lower. Though in participants without CVD history, LDL was significantly lower in opium users compared to non-users, there was no difference in participants with CVD history. However, since higher LDL is related to CVD, this could be due to residual confounding or selection bias.

In present study, consumption duration of opium above the mean resulted in lower odds of high TC and high LDL disorders. Dose of opium consumption more than mean resulted in the lowest odds of dyslipidemia, high TC, and high LDL disorders in the full adjusted model. In the case of consumption route, opium smoking showed an inverse relation with high TC and high LDL disorders and a positive relation with low HDL disorder. Also, oral administration was a significant conservative factor for dyslipidemia and high TC. In study of Asgary et al. HDL was lower in opium users and the routes of opium consumption (smoking or oral) had no effect on its outcomes. In their study, when the duration of opium usage increased, no significant differences were observed in TC, TG and HDL levels. However, the duration of addiction more than 2 years significantly increased LDL^33^. Hosseini et al. observed a dose–response relationship between dose of opium with severity of coronary artery diseases after adjustment for potential confounders. They showed no significant differences between the routes of opium consumption (smoking or oral) regarding the extent and severity of coronary artery diseases^36^. Furthermore Mohammadi et al. showed that oral administration of opium caused an increase in the levels of TC, TG and LDL and accelerated atherosclerosis formation in hypercholesterolemic rabbits^37^. In another study by the authors on hypercholesterolemic rabbits that were exposed to opium by smoking, HDL decreased and a non-significant relation on the other lipid components was seen^38^. Therefore, the route of opium consumption might have affected the effect of opium on lipid profile. Opium is poorly absorbed in the stomach but well absorbed in the small intestine. In contrast, vaporized morphine produced by smoking of opium is rapidly absorbed in the lungs into the bloodstream, and within a few seconds is available at the brain. Hence the onset of action is more rapid after smoking, but the duration of action is longer after oral ingestion^39^.

Contradiction in these results could be due to different conditions between addicts and non-addicts. Some of these factors include: (1) Nutritional factors: such as loss of appetite, malnutrition and vitamin deficiency in addicted subjects^40^. Therefore, the lower lipids in opium users in some studies might be due to weight loss or unhealthy diet not owing to the direct effect of opium. (2) Individual factors such as underlying diseases, psycho-social problems, physical activity intensity, and age range of participants. (3) Factors relevant to the opium including its purity and ingredients, concomitant use of other substances (alcohol, cigarette, tobacco,…), variations in dose, duration and route of opium consumption in different studies. (4) The factors relevant to research: including sample size or hospital-based study instead of population-based study. Therefore, the outcomes may not be generalized to the whole population.

Regarding the mechanism of the effects of opium on lipid profile, some probable mechanisms proposed are reduction of hepatic clearance of LDL from the plasma and induction of hepatic synthesis of TG, leading to increased levels of TC and TG respectively^41^. Opium is mainly metabolized in the liver. Chronic use of morphine causes the suppression of liver antioxidant system, elevation of oxidant factors and induction of apoptosis in liver cells in mice model^42^. There is a direct association between the duration of heroin consumption and the severity of liver injury in heroin abusers^43^. Since the liver plays a pivotal role in the metabolism of lipids, chronic liver diseases are associated with significant alterations in serum lipids^44^. Maccaria et al. indicated that TG increased while TC and HDL decreased in heroin addicts and showed an inverse relation between TC and alanine aminotransferase (ALT), an indicator of liver damage. Accordingly, the authors concluded that lower TC might be due to liver damage which is common in heroin users^45^. In addition morphine affects the metabolism in different ways by increasing hormones such as adrenalin, noradrenaline, corticosterone, prolactin and glucagon^46^.

The importance of serum lipid profile is known as a risk factor of cardiovascular diseases. Despite lower levels of TC, and LDL in opium users in comparison with non-users in our study, opium usage is not recommended for decreasing lipid profile and risk of heart disease. Our results showed that the frequency of CVD history in opium users was significantly higher than non-users. Also, most current evidence showed the increased risks of ischemic heart disease, heart attack^15^, hypertension^47^ and cancer^48^ in opium users. According to study of Ziaee et al. although opium consumption ralatively decreases LDL and TC levels, increasing the dose and duration of this compound in the long-term can cause changes in plasma fibrinogen levels, clotting, and atherosclerosis and also develop coronary artery diseases, hypertension, and stroke in patients^49^. Therefore, healthcare managers and patients should be aware of the side effects of opium consumption on various vascular events. In addition, it is necessary for healthcare managers to raise the level of awareness and health literacy of the general public about the harmful effects of opium use and to take effective strategies to prevent and reduce opium usage.

## Strengths of the study

As the strength of our study, there is the allowance for several confounding factors, including BMI, physical activity, history of underlying diseases influencing dislipidemia, fat intake of the diet and use of hepatotoxic drugs. The large sample size and a population-based research are other main strengths of our research. Furthermore, we used one type of device and a single laboratory to measure the lipid profile in whole participants. Another strength of our study was evaluation of the association between lipid disorders and dose of opium usage. However, the study has some limitations. Data of opium use based on self-reporting may cause misreporting when compared with biological tests, since it is possible that some people did not answer the opium use questionnaire correctly. For this reason, these studies are susceptible to measurement errors such as self-reporting bias and recall biases, which may result in some deviations from reality (incidence of bias in estimates)^50–55^. However, the amount of this bias depends on geographical area and the population under study^55^. We believe that the validity of self-reported opium use over the past years in our population, especially in the adult population is relatively high, due to low social stigma for opium use in this population^55,56^.

## Conclusion

Despite variable impact on serum lipids, opium has known side effects on many organs and increases risks of ischemic heart disease, heart attack, hypertension and cancer.

## Supplementary Information

Supplementary Table 1.

## Data Availability

The datasets used during the current study are available on the Persian Adult Cohort Study Center, Rafsanjan University of Medical Sciences, Iran. The data is not available publicly. However, upon a reasonable request, the data can be obtained from the authors.
