# Supplementary material for: Prevalence of dyslipidemia and its association with opium consumption in the Rafsanjan cohort study
Source: Sci Rep. 2022 Jul 7;12:11504. doi: 10.1038/s41598-022-13926-3 (PMC9262952; doi:10.1038/s41598-022-13926-3)
Supplement: Supplementary file 1 — Supplementary Table 1. [file 41598_2022_13926_MOESM1_ESM.docx]

| **eTable 1. Association of opium consumption with dyslipidemia, High TC, High TG, Low HDL and High LDL in participants with and without CVD history (n= 9932).** | | | | |
| --- | --- | --- | --- | --- |
| **Variables** | **Subjects without CVD(n=8903)** | | **Subjects with CVD(n=1029)** | |
|  | **Crude model OR (95%Ci)^a^** | **Adjusted model OR(95%Ci)^b^** | **Crude model OR (95%Ci)^a^** | **Adjusted model OR (95%Ci)^b^** |
| **Dyslipidemia** | |  |  |  |
| **Opium consumption** | |  |  |  |
| no | 1 | 1 | 1 | 1 |
| yes | 0.78(0.70-0.87) | 0.94(0.80-1.09) | 0.55(0.38-0.79) | 0.43(0.24-0.76) |
| **High TC** | |  |  |  |
| **Opium consumption** | |  |  |  |
| no | 1 | 1 | 1 | 1 |
| yes | 0.69(0.63-0.77) | 0.83(0.71-0.95) | 0.65(0.48-0.87) | 0.54(0.35-0.84) |
| **High TG** |  |  |  |  |
| **Opium consumption** |  |  |  |  |
| no | 1 | 1 | 1 | 1 |
| yes | 1.16(1.05-1.28) | 1.04(0.90-1.20) | 1.02(0.79-1.33) | 1.45(1.01-2.10) |
| **Low HDL** |  |  |  |  |
| **Opium consumption** |  |  |  |  |
| no | 1 | 1 | 1 | 1 |
| yes | 0.61(0.51-0.73) | 1.27(0.98-1.63) | 0.72(0.51-1.03) | 1.11(0.66-1.84) |
| **High LDL** |  |  |  |  |
| **Opium consumption** |  |  |  |  |
| no | 1 | 1 | 1 | 1 |
| yes | 0.68(0.61-0.76) | 0.76(0.65-0.89) | 0.82(0.62-1.07) | 1.03(0.55-1.27) |
